# The Effect of the Presence of Amino Acids on the Precipitation of Inorganic Chemical-Garden Membranes: Biomineralization at the Origin of Life

**DOI:** 10.1021/acs.langmuir.2c01345

**Published:** 2022-08-17

**Authors:** Ana Borrego-Sánchez, Carlos Gutiérrez-Ariza, C. Ignacio Sainz-Díaz, Julyan H. E. Cartwright

**Affiliations:** † Instituto Andaluz de Ciencias de la Tierra (CSIC-University of Granada), Armilla, 18100 Granada Spain; ‡ Department of Pharmacy and Pharmaceutical Technology, Faculty of Pharmacy, University of Granada, Campus de Cartuja s/n, 18071 Granada, Spain; § Instituto Carlos I de Física Teórica y Computacional, Universidad de Granada, 18071 Granada, Spain

## Abstract

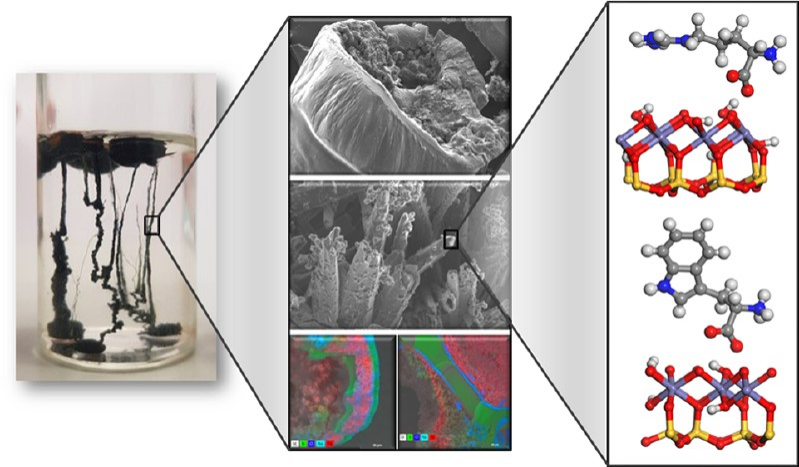

If life developed in hydrothermal vents, it would have been within mineral membranes. The first proto-cells must have evolved to manipulate the mineral membranes that formed their compartments in order to control their metabolism. There must have occurred a biological takeover of the self-assembled mineral structures of the vents, with the incorporation of proto-biological molecules within the mineral membranes to alter their properties for life’s purposes. Here, we study a laboratory analogue of this process: chemical-garden precipitation of the amino acids arginine and tryptophan with the metal salt iron chloride and sodium silicate. We produced these chemical gardens using different methodologies in order to determine the dependence of the morphology and chemistry on the growth conditions, as well as the effect of the amino acids on the formation of the iron-silicate chemical garden. We compared the effects of having amino acids initially within the forming chemical garden, corresponding to the internal zones of hydrothermal vents, or else outside, corresponding to the surrounding ocean. The characterization of the formed chemical gardens using X-ray diffraction, Fourier transform infrared spectroscopy, elemental analysis, and scanning electron microscopy demonstrates the presence of amino acids in these structures. The growth method in which the amino acid is initially in the tablet with the iron salt is that which generated chemical gardens with more amino acids in their structures.

## Introduction

In the period immediately prior to the dawn of life on Earth, chemical reactions must have been producing amino acids, the building blocks of more complex organic molecules, proteins, and nucleic acids, that would go on to form the first proto-cells; the first life.^1^ One leading theory is that life on Earth emerged at submarine alkaline hydrothermal vents in the oceans more than 4 billion years ago.^2−4^ At these hydrothermal vents, mineral-laden alkaline water emerges into the surrounding ocean of a different pH and temperature, depositing its mineral load as inorganic precipitates that form highly complex, intricate structures at the vent. Laboratory analogues of these geological structures have been known and grown for centuries in the chemical laboratory; owing to their resemblance to plants they are known as chemical gardens.^5,6^

Within some of the pores of the hydrothermal vent structures, the pH, temperature, pore size may be “just right” for a complex proto-biochemistry to be able to begin to self-organize, so that the first proto-cells might be found within these hydrothermal complexes.^7^ If life first incubated in mineral membranes within the hydrothermal vents, there must have occurred a biological takeover of the self-assembled mineral structures in the first proto-cells, with the incorporation of proto-biological molecules within the mineral membranes to alter their properties for life’s purposes. The first proto-cells must have evolved to manipulate the mineral membranes that formed their compartments in order to control their metabolism. For these reasons, it is important to know what effect the presence of amino acids has on the precipitation of inorganic chemical-garden membranes.

Two representative examples of amino acids that are essential in the building of the components of life are arginine and tryptophan. We selected a short polar amino acid (arginine) and a larger aromatic amino acid (tryptophan) to compare amino acids with different size, polarity, and electronic structure. Arginine is considered in adult humans to be a conditionally essential amino acid because it is usually produced in adequate amounts by endogenous synthesis (nonessential), but it is also required exogenously under certain circumstances (illness and stress). However, it is also considered an essential amino acid in birds, carnivores, and young mammals.^8^ It is involved in the synthesis of important compounds for living organisms, such as creatine and polyamine. Previous studies have also detailed the fundamental role that arginine has in the synthesis of peptides and nucleic acids. Specifically, the interactions between arginine and nucleic acids, the electrostatic interactions generated between the positive charge of arginine and RNA, represent the first steps in the evolution of the genetic code of living systems.^9,10^ Tryptophan is considered an essential amino acid for normal growth of young animals,^11,12^ and it is also necessary for the maintenance of nitrogen equilibrium in mature animals.^13,14^ Tryptophan, classified as a nonpolar hydrophobic amino acid, is one of the amino acids expressed in the genome of most living beings. It is involved in protein synthesis and is a precursor of biologically active compounds and important coenzymes, playing a crucial role in many metabolic functions.^15^

Previous works have highlighted the role of iron oxide-hydroxide minerals in prebiotic chemistry and the origin of life.^2,3,16^ In the framework of the interaction between amino acids and minerals in chemical gardens, previous works have studied the synthesis of alanine in iron oxyhydroxide mineral systems,^16^ the presence of other amino acids, such as glycine, alanine, cysteine, aspartate, and lysine, in iron-silicate chemical gardens,^17^ and the effects of cysteine, histidine, and arginine on phosphate adsorption onto iron (oxy)hydroxide minerals.^18^ However, these works have not looked at what is arguably the more pertinent case to the origin of life that we study here: when the amino acid is present initially within the forming chemical-garden structure.

In this work, the interaction and the adsorption processes of arginine and tryptophan amino acids in the formation of iron-silicate chemical gardens are studied. These chemical gardens were formed under distinct growth conditions. To determine the morphology and chemistry and the effect of the amino acids in the formation of the iron-silicate chemical garden, several techniques were used: macrophotography, X-ray diffraction, Fourier transform infrared spectroscopy, elemental analysis and environmental scanning electron microscopy.

## Materials and Methodology

### Materials

l-Arginine, L-tryptophan, iron(II) chloride tetrahydrate (FeCl_2_·4H_2_O), and sodium silicate (6.25 M) were purchased from Sigma-Aldrich. Deionized water was used in all experiments.

### Methodology

#### Formation of Chemical Gardens

Chemical gardens of the soluble metal salt iron(II) chloride and the amino acids arginine and tryptophan were formed in a solution of sodium silicate by using different methodologies.

**Figure 1:**
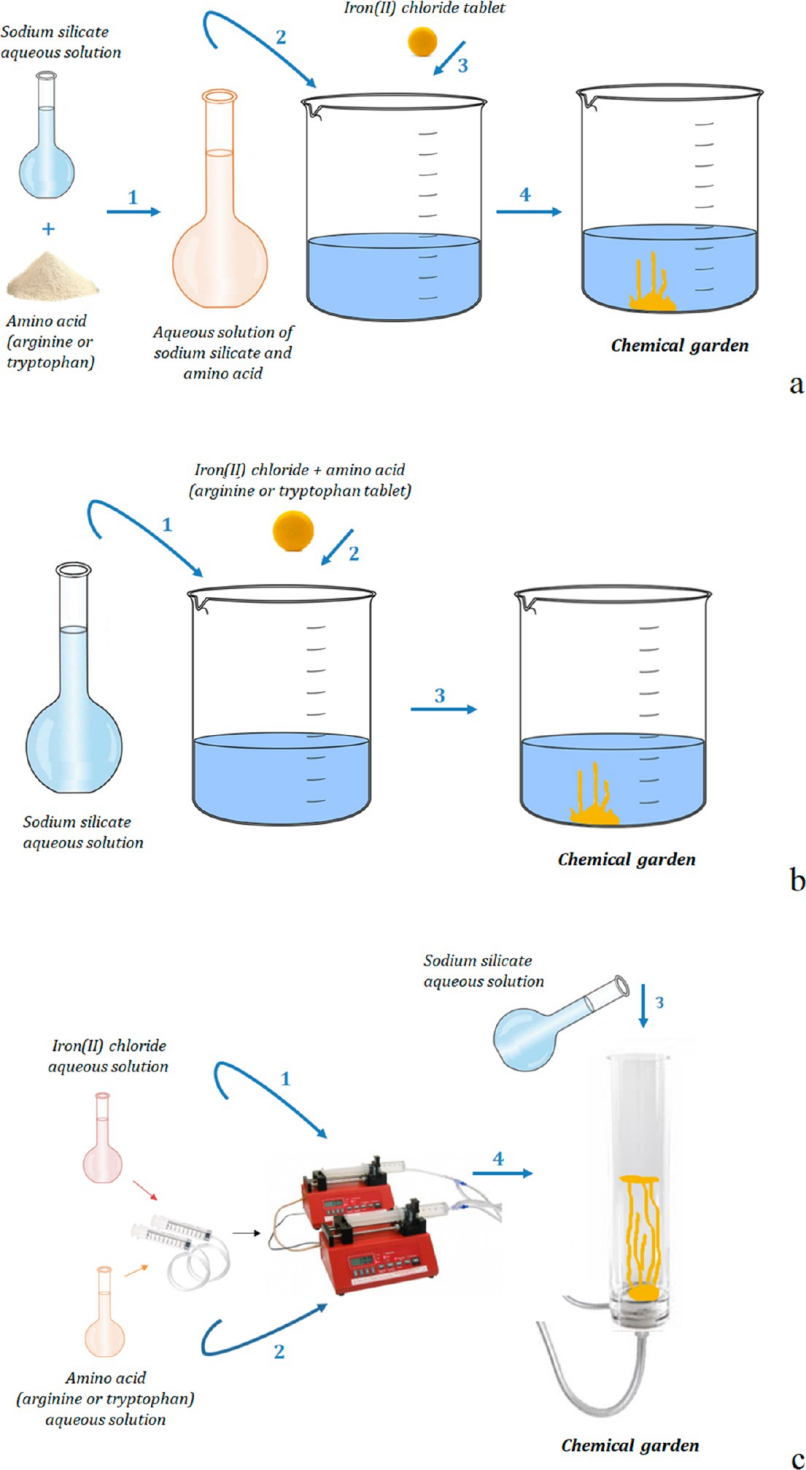
Methodologies of chemical-garden formation with the amino acid initially in solution (a), in a tablet (b), or introduced by injection (c).

In the first one, the solution method, 696.8 mg of arginine (0.2 M) was dissolved under agitation in 20 mL of an aqueous solution 4.22 M of sodium silicate. In a similar fashion, 81.7 mg of tryptophan (0.02 M) was dissolved in 20 mL of 4.22 M aqueous solution of sodium silicate. Crystals of iron(II) chloride were pulverized in an agate mortar and 200 mg was pressed into cylindrical tablets of 5 mm diameter and 1 mm height using a Specac Manual Hydraulic Press with 2 bar of pressure during 5 min. This step was designed to avoid having initial conditions of different shapes and to obtain a systematically uniform composition and shape. The freshly prepared iron chloride tablet was added into the solution with sodium silicate and the amino acid (Figure 1a).

The second method was the tablet method, 200 mg of iron(II) chloride and 696.8 mg of arginine or 81.7 mg of tryptophan homogeneously mixed in an agate mortar were used and pressed into cylindrical tablets of 5 mm diameter and 1 mm height using a Specac Manual Hydraulic Press with 2 bar of pressure during 5 min. Each freshly prepared tablet of the metal salt and amino acid was added into 20 mL of 4.22 M aqueous sodium silicate solution (Figure 1b).

Lastly, an injection method was used.^19−22^ For this, 40 mL of sodium silicate solution of 4.22 M concentration was prepared and placed in a cylindrical glass container. Crystals of iron(II) chloride and the amino acids were pulverized with an agate mortar. 200 mg of iron(II) chloride was dissolved in 5 mL of water, and the same amount of amino acid as in the previous methods above was used, 696.8 mg of arginine and 81.7 mg of tryptophan were dissolved in 5 mL of water, respectively in these initial solutions. The freshly prepared solutions of iron chloride and amino acid were injected simultaneously into the aqueous solution of sodium silicate using two LA-120 syringe pumps at 80 mL/h and 50 mL/h flow rates, respectively (Figure 1c).

#### Characterization of Chemical Gardens

The chemical gardens formed were characterized with different techniques.

##### Macrophotography

Photographs of the chemical gardens formed were taken with a Nikon reflex camera with a macrolens.

##### X-ray Diffraction

Powder X-ray Diffraction (XRD) analyses were performed in a PANalytical X’Pert PRO diffractometer. The samples were analyzed directly using a Bruker D8 DISCOVER diffractometer with microfocus beam of variable diameter (0.1–2 mm) at a wavelength of 1.54 Å, and a DECTRIS PILATUS3R 100 K-A detector. The identification of crystallographic phases in the diffractograms was performed with the Xpowder code.^23^

##### Fourier Transform Infrared Spectroscopy

Fourier transform infrared spectroscopy (FTIR) spectra of the samples studied were recorded in the range 4000–600 cm^–1^ with 0.5 cm^–1^ resolution and a well-plate sampler. The spectra were obtained with a JASCO 6200 spectrophotometer and analyzed with Spectra Manager II software.

##### Elemental Analysis

Elemental analyses of the samples for the determination of carbon, hydrogen, and nitrogen components were carried out with a Thermo Scientific Elemental Analyzer Model Flash 2000.

##### Scanning Electron Microscopy

The micrographs of the samples were obtained using a FEI Quanta 400 environmental scanning electron microscope (SEM) at high vacuum and room temperature for the silicate experiments. Chemical analysis of the solid surfaces was performed in situ in the microscope using energy dispersive X-ray spectroscopy (EDX) analysis.

## Results and Discussion

### Macrophotography

**Figure 2:**
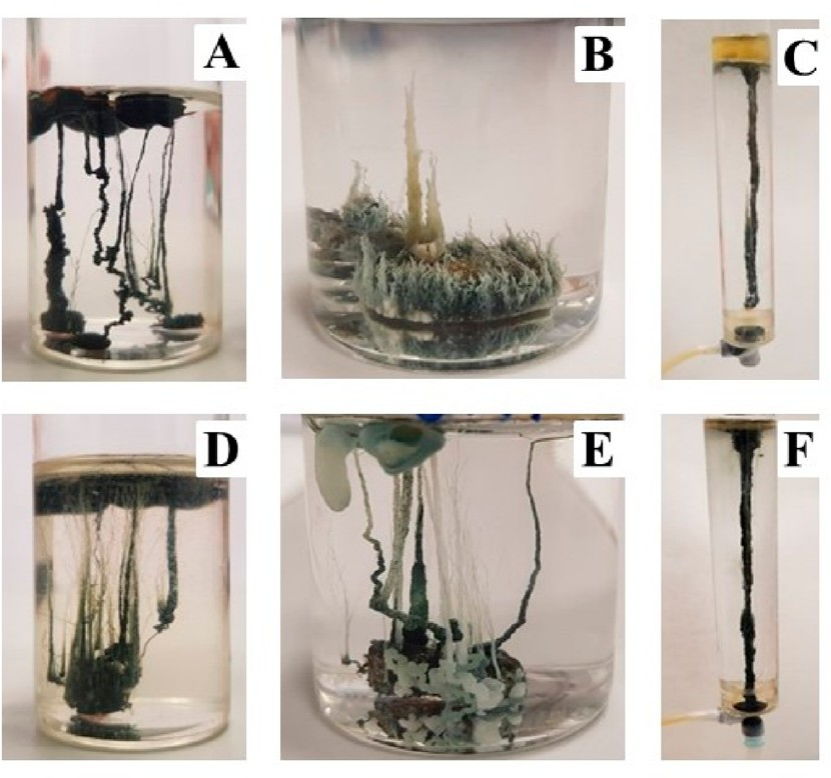
Images obtained of the chemical gardens formed with A: arginine initially in solution; B: arginine initially in tablet; C: arginine solution injected; D: tryptophan initially in solution; E: tryptophan initially in tablet; and F: tryptophan solution injected. All reactors have 3 cm of inner diameter.

In the case of the chemical garden in which the metal salt is in the form of a tablet, both with amino acids in solution (Figure 2A,D) and also with amino acids in the tablet (Figure 2B,E), a semipermeable membrane was generated around the tablet, swelling under osmosis with water from the external solution and increasing its volume. In most cases, this internal pressure produced some breaks in this membrane and the jets of the internal fluid exited upward owing to their buoyancy. In the first stages, these fluid jets did not produce precipitation, but later some precipitates were deposited in the interface of these jets with the external fluid. The chemical gardens shown in Figure 2A,D,E formed one or more bulbs from which many long tubes of different thicknesses emerged. Some of the tubes were smoother and others generated spiral shapes. Figure 2B, in which the arginine was initially in the tablet, showed the formation of a different type of chemical garden. From the large bulb, a multitude of very thin and short tubes was formed.

In the case of injection growth, the injection of the iron chloride solution was carried out simultaneously and at a higher velocity than the slow injection of an amino acid solution. With both amino acids, the formation of chemical-garden structures was similar. A single tube was formed, which is very tall, compact, thick, and without small tubes around it (Figure 2C,F). We tried to minimize the risk of oxidation of Fe(II), and no red color of Fe(III) systems was observed (Figure 2). Nevertheless, this effect is not critical for our results.

### X-ray Diffraction

**Figure 3:**
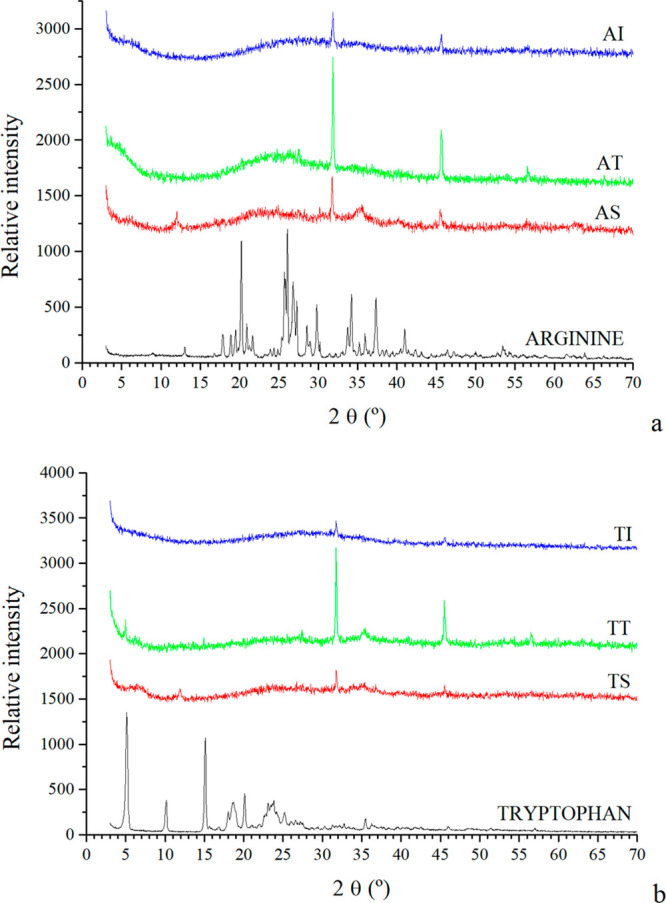
Diffractograms of the chemical gardens with arginine (a): AS: arginine initially in solution; AT: arginine initially in tablet; AI: arginine by injection; and with tryptophan (b): TS: tryptophan initially in solution; TT: tryptophan initially in tablet, and TI: tryptophan by injection.

The results of XRD allowed characterizing the composition of the chemical gardens (Figure 3). The diffractogram of pristine arginine showed the most intense peaks at 20.2° and 26°, as well as other peaks at 25.6°, 26.8°, 27.2°, 29.8°, 34.2°, and 37.3°. In the diffractograms of the chemical gardens generated with arginine, the peaks of the amino acid are not clearly observed because of the amorphous state of the precipitated amino acid in the chemical garden and/or to the low amount of arginine in them (Figure 3a). This can be appreciated in the broad peak at 18–30° in the solids formed with arginine included in the initial tablet.

The diffractogram of pristine tryptophan showed its two most intense peaks at 5.1° and 15.1°. These peaks can just be observed in the sample in which the tryptophan was initially in the tablet, while they are not detected in the rest. This indicates that the sample with the tryptophan initially in the tablet incorporates a higher amount of amino acid than the other samples with tryptophan (Figure 3b).

In all cases, the formation of highly crystalline halite (NaCl) was observed with reflections at 32° and 46° (2θ units). No peaks related to Fe(II) chloride were detected.^24^ Other peaks are detected and can be assigned to Fe hydroxides at 34–36° and 62–63°,^25^ especially in the chemical gardens generated with arginine in solution. These reflections can be seen also in samples generated with tryptophan in the tablet and in solution.

### Fourier Transform Infrared Spectroscopy (FTIR)

**Figure 4:**
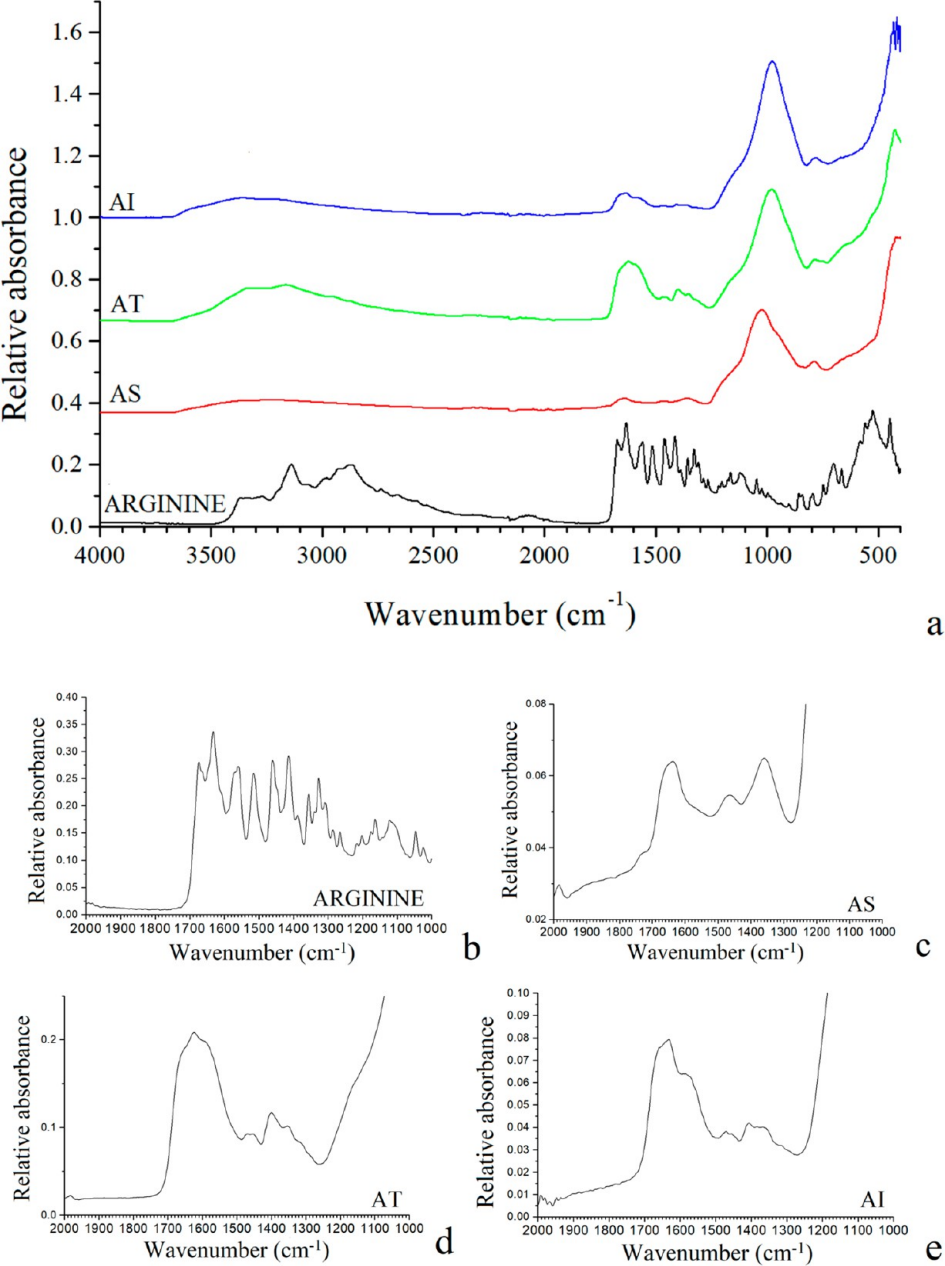
FTIR spectra of the chemical gardens with arginine (a), highlighting the 1800–1200 cm^–1^ range (b–e). AS: arginine initially in solution; AT: arginine initially in tablet; and AI: arginine by injection.

**Figure 5:**
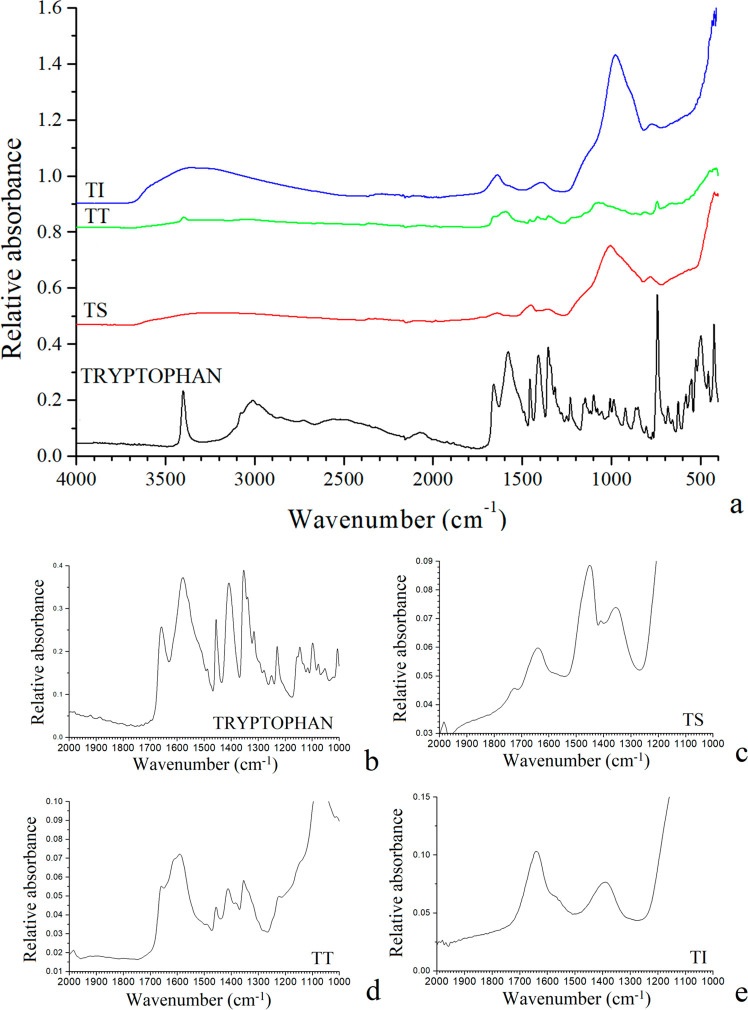
FTIR spectra of the chemical gardens with tryptophan (a) zooming into the 1800–1200 cm^–1^ range (b-e). TS: tryptophan initially in solution; TT: tryptophan initially in tablet; and TI: tryptophan by injection.

**Figure 6:**
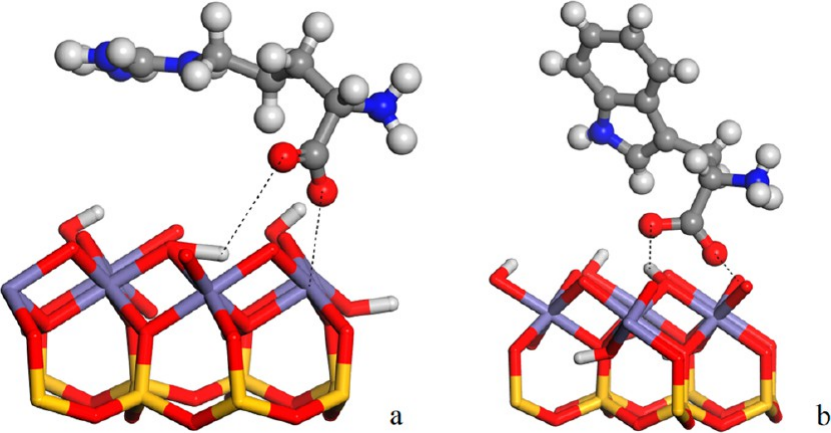
Molecular structure models of possible interactions of arginine (a) and tryptophan (b) with the iron silicate solid surface. Distances are in Å. The atoms in red, clear-gray, gray, blue, fuchsia, and yellow represent the O, H, C, N, Fe, and M (Si or Fe) atoms.

The FTIR spectra of the samples studied are shown in Figures 4 and 5. Specifically, the infrared spectrum of arginine revealed the most intense band in the ranges of 3500–2500 cm^–1^ and 1700–1250 cm^–1^. The chemical gardens generated with arginine indicated some characteristic bands of pristine arginine, which shows that in all cases there is arginine incorporated in the structure of the chemical garden. In the chemical garden produced with arginine initially in the tablet, the typical bands from the amino acid have more intensity, indicating a greater amount of amino acid (Figure 4). We can observe that the profile of arginine spectra changes in the 1800–1300 cm^–1^ range after its absorption on the chemical-garden surfaces. The ν(C=O) stretching band at 1674 cm^–1^ of the pristine arginine disappears after the absorption and shifts to lower frequencies at 1342 cm^–1^ in AS. This indicates that the carboxylic group has become a carboxylate group by forming a complex with a metal oxide of the chemical-garden surface. In other words, a chemical adsorption of arginine has occurred in a zwitterionic form. This change is consistent with previous spectroscopic studies of the formation of arginine-Fe complexes.^26^ However, the multiband at 1650–1600 cm^–1^ corresponding tentatively to the stretching ν(C=N) (at 1622 cm^–1^)^26^ of the guanidine moiety CN_3_H_5_^+^; the bands at 1559 and 1515 cm^–1^ corresponding to the bending δ(NH_2_) mode; and the band at 1460 cm^–1^ assigned to δ(CH_2_),^27^ remain without significant alteration after absorption and only a broadening effect is observed (Figure 4). However, in AS the intensity of the bands at 1570–1480 cm^–1^ decreases significantly when arginine is adsorbed on the solid surface. The band at 1414 cm^–1^ assigned to symmetric ν(C=O) mode of carboxylate moiety is shifted slightly to lower frequencies. These changes in frequency and relative intensity of the IR bands with amino acid adsorption are different in each case of the chemical-garden formation method used in this work. This may be due to the formation of different complexes between the carboxylate group and the surface atoms of solid, for instance, the carboxylate O atoms can coordinate directly the Fe cation or form hydrogen bonds with the hydroxide groups of the Fe-oxy-hydroxide moiety (Figure 6).

The tryptophan amino acid spectrum revealed intense bands in the ranges of 3500–2800 cm^–1^ and 1700–1300 cm^–1^. These bands are also observed in the formed chemical gardens, confirming the presence of tryptophan in these structures (Figure 5). In TS, the relative intensities of the tryptophan bands change in TI, TT, and TS, indicating an interaction between tryptophan and Fe cations. This interaction seems to be different in each case and also different to that of pristine tryptophan.

In the spectrum of solid pristine tryptophan, the intense band at 3398 cm^–1^ corresponds to the stretching ν(NH) vibration mode of the heterocyclic NH bond in the molecular packing in the crystal lattice.^28^ This band disappears in the solids formed with tryptophan, which we can interpret as the crystals of tryptophan being dissolved and reprecipitated as an amorphous phase along with chemical gardens. The ν(C=O) stretching band at 1657 cm^–1^ in tryptophan shifted to lower frequencies in all absorption cases because of the formation of tryptophan-metal oxide complexes on the surface of the chemical garden (Figure 6), according to previous IR studies of metallic complexes with tryptophan.^29^ The bands at 1456 cm^–1^ in tryptophan can be assigned to a δ(CH_2_) mode, and no significant change was observed with the adsorption process.^30^ The bands observed at 1408, 1353, 1339, and 1315 cm^–1^ can be assigned to ν_*s*_(O–C–O^–^), ν(C–N)_indol_ and δ(CH) modes, respectively. Bands corresponding to pristine tryptophan are detected in TT, indicating that some portion of this amino acid remains, not being chemically adsorbed onto the solid surface, corroborating that observed in XRD (see above). However, the band observed in tryptophan at 1578 cm^–1^, assigned tentatively to δ(NH_2_), is not detected in TS, and at the same time the band at 1720 cm^–1^ in TS could indicate tentatively a shift of the δ(NH_2_) band to higher frequencies due to the participation in the coordination of tryptophan with the solid surface. This phenomenon was observed previously in the formation of organic complexes with metal cations.^31^ Hence, our results confirm that tryptophan is chemisorbed on the chemical-garden surfaces.

### Elemental Analysis

The mean of the results of at least three measurements are shown in Table 1. These results show that the chemical gardens prepared with arginine have a higher percentage of nitrogen, taking into account the chemical compositions of the amino acids. This may indicate that a higher initial amount of amino acids in turn implies that a greater amount of amino acid is present in the chemical garden formed. In addition, the use of one or another amino acid can vary the presence of amino acids in the chemical garden (Table 1). Chemical gardens prepared following the second methodology, in which the amino acid is initially with the iron salt in the tablet and the sodium silicate solution around it, are those in which the greatest amount of amino acid is present in the assembled chemical garden. The injection method is the next method that retains more amino acid quantity in the chemical garden. The solution method where the amino acid is dissolved in the sodium silicate solution is that in which the chemical garden has the least amino acid amount (Table 1). In some cases, the amount of C is high due to a certain carbonate precipitation.

**Table 1:** Average of the Percentage of Nitrogen, Carbon, and Hydrogen in the Studied Samples^a^

sample	% N	% C	% H
AS	0.763 (±0.012)	2.553 (±0.026)	1.710 (±0.051)
AT	10.647 (±0.252)	13.913 (±0.242)	3.970 (±0.236)
AI	2.565 (±0.018)	3.898 (±0.025)	2.255 (±0.109)
TS	0.077 (±0.009)	2.067 (±0.063)	1.670 (±0.045)
TT	3.100 (±0.033)	14.680 (±0.116)	2.500 (±0.099)
TI	0.430 (±0.007)	3.128 (±0.311)	2.545 (±0.647)

a AS: arginine initially in solution; AT: arginine initially in tablet; AI: arginine by injection; TS: tryptophan initially in solution; TT: tryptophan initially in tablet; and TI: tryptophan by injection. Standard deviation is shown in parentheses.

### Scanning Electron Microscopy (SEM)

**Figure 7:**
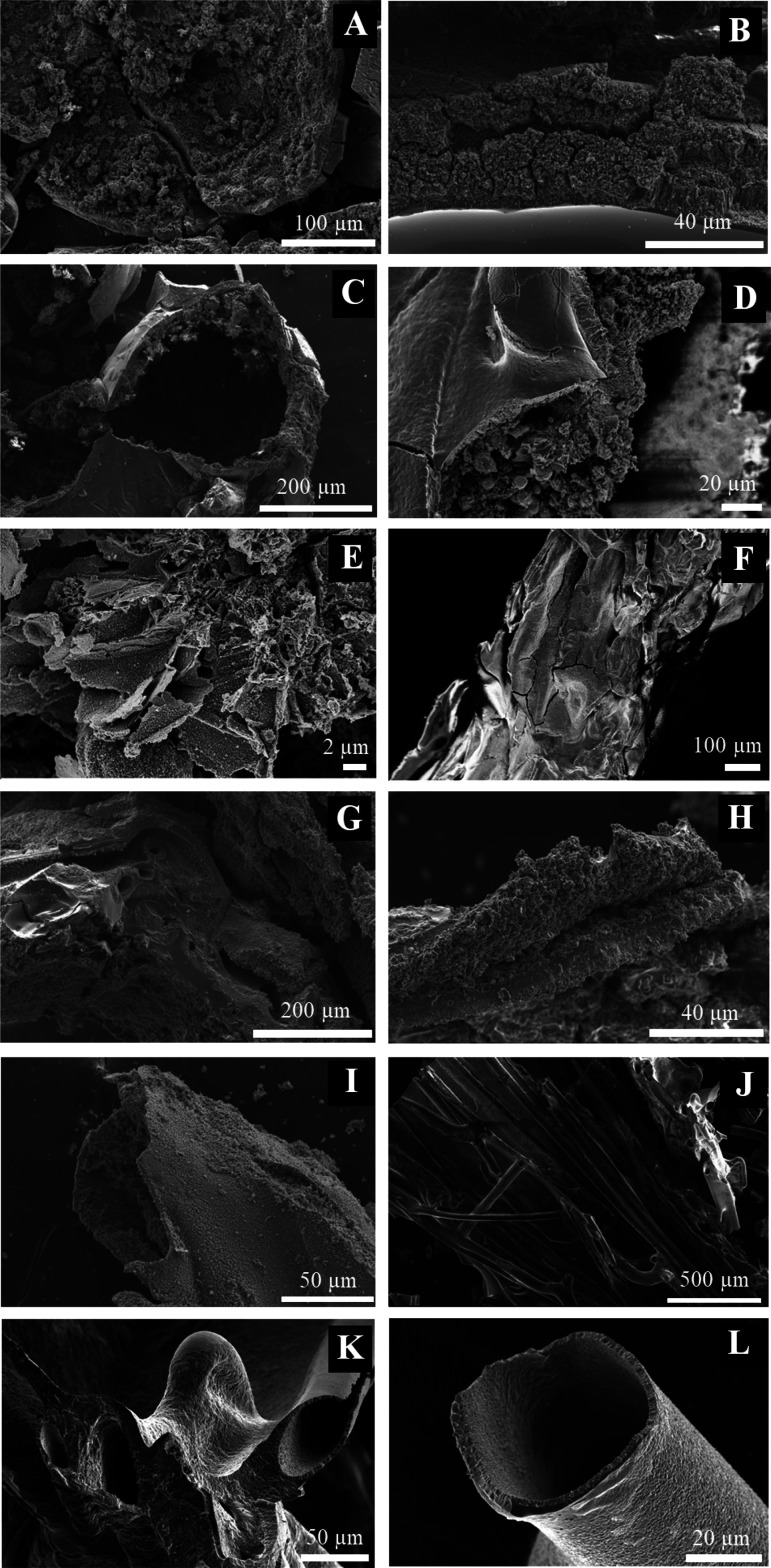
SEM micrographs of samples formed with arginine in solution (A–E), in tablets (F,G), by injection method (H,I), and controls without amino acid (J–L).

**Figure 8:**
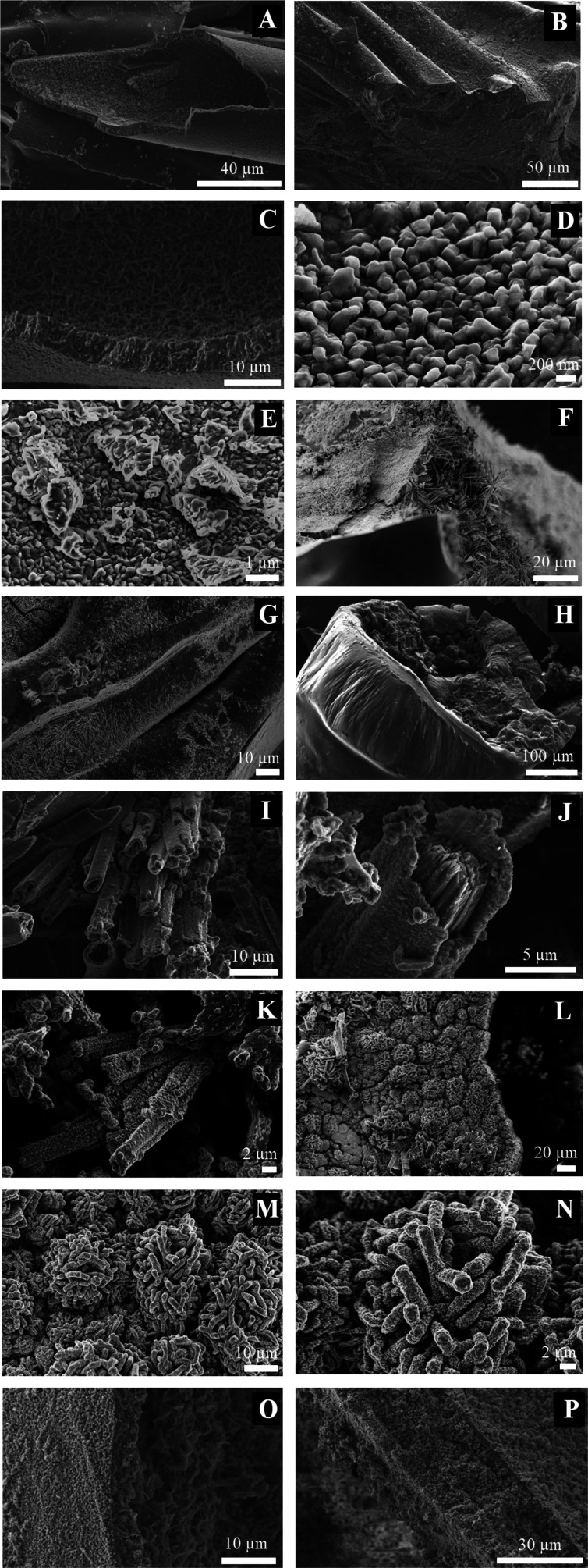
SEM micrographs of samples formed with tryptophan in solution (A–G), in tablet (H–N), and by injection (O,P).

The samples of chemical gardens were observed microscopically with SEM (Figures 7 and 8). Different crystal structures are formed depending on the addition method of amino acid into the reaction and also depending on the amino acid itself. In tubes formed with arginine and tryptophan in solution, an external smooth surface and a rough internal one is observed. In the case of the chemical gardens with arginine initially in the tablet, the morphology is different. A smooth layer is found between two grainy layers (Figure 7F,G). In the case in which arginine is injected, the structure formed is more disordered. An internal layer with round structures is observed, and there are two external layers that are not completely smooth; they also have round structures on the surface (Figure 7H,I).

In the chemical gardens with tryptophan (Figure 8), the microsurfaces differ, indicating that the presence of amino acids affects the chemical-garden formation. When tryptophan is initially in solution, a bilayer structure is observed as with arginine. However, the external membrane is not completely smooth as in the case of the arginine; it has a porous structure. In addition, the internal surface is formed by different porous structures, looking like hairs (Figure 8A–G). When the tryptophan is initially in the tablet, the morphology is different. An external smooth membrane is observed. The interior is rough, with structures like balls and small tubes with other forms, elongated structures or flower-like forms inside (Figure 8H–N). Lastly, in the case in which tryptophan was injected, the structure formed is disordered, as with arginine. The morphology shows very thin smooth layers and most of the structures are porous and circular (Figure 8O,P).

As a control test, chemical gardens grown without amino acid the morphology and surface characteristics differ with respect to those formed with amino acids (Figure 7J–L).

For the injection experiments no such clearly separated layer structure is found. All compounds are mingled in a more chaotic way, likely due to the injection rates not matching the adsorption rates of the amino acid into the chemical gardens, which seems to be a more decisive factor than the presence or absence of the amino acid itself. This latest result, although not determinant, encourages some further research with varying injection rates to shed more light on how and how fast the amino acids might affect the growth of chemical gardens.

**Figure 9:**
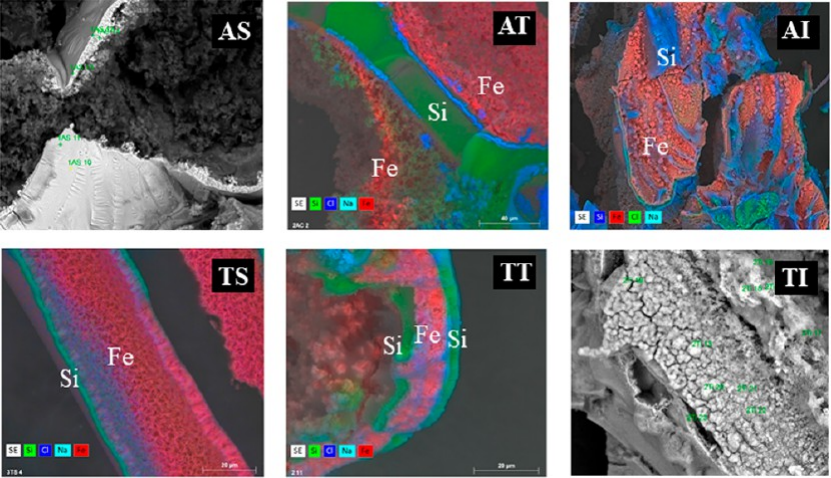
EDX microanalysis graphs and mappings of samples formed with arginine in solution, mainly Fe and silicate and Fe oxides (AS), in tablets (AT) and by injection method (AI); and formed with tryptophan from solution (TS), in tablets (TT) and injection (TI).

**Figure 10:**
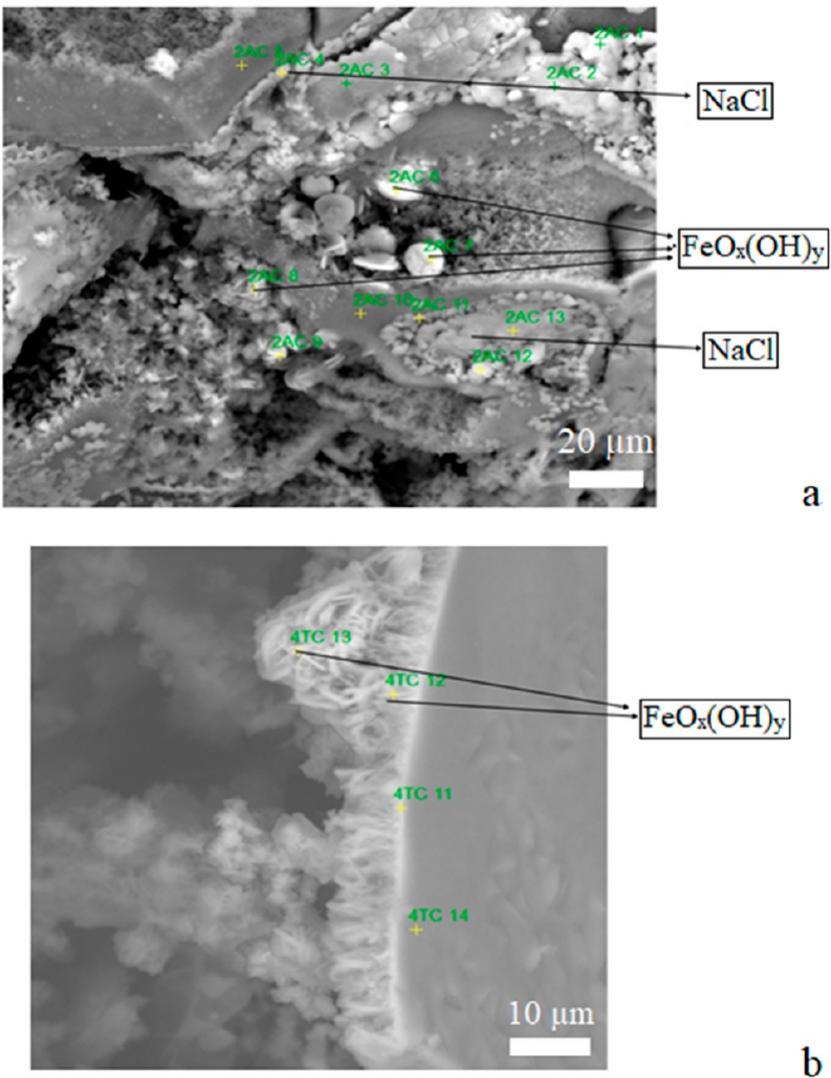
Crystals of Fe oxide in AT (a) y TT (b).

The chemical analysis of the external surface shows that it is formed predominantly of Fe and Si with Fe oxide/hydroxide with small crystals of NaCl. In other interface zones, some crystals with a platelet morphology composed mainly of Fe oxide/hydroxide can be observed (Figures 9 and 10).

In summary, it is observed that the experimental method in which the amino acid is initially in solution gives rise to bilayer structures. When the amino acid is initially in the tablet the structure is trilayered. The injection methods present more disordered structures. In general, the smooth layers are formed mainly of silicon, and the porous layers are formed mainly of iron (Figure 9). However, the composition and morphology of these layers vary with each amino acid.

**Figure 11:**
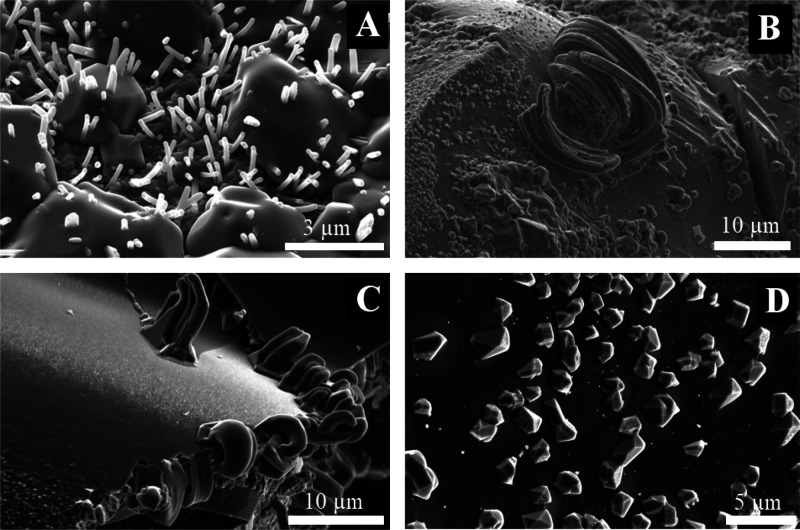
Crystal growth of materials rich in halide salts, with (A,B) and without (C,D) amino acids.

Additional crystal structures are observed formed by halide salts, mainly sodium chloride. These structures (Figure 11) show the tendency of the crystal growth to follow the trajectory of Na^+^ cation diffusion from the external solution crossing the osmotic membrane through a chloride rich environment.

## Conclusions

The formation of iron(II)-silicate chemical gardens in the presence of the amino acids arginine and tryptophan has been investigated to study how amino acids affect the growth of iron(II)-silicate chemical gardens. With different techniques, including X-ray diffraction, Fourier transform infrared spectroscopy, elemental analysis and scanning electron microscopy, we analyzed the inorganic precipitates formed. The results demonstrated the presence of the amino acid in the structure of these chemical gardens. Specifically, a higher concentration of the amino acids in the chemical gardens was obtained when the amino acids were initially placed in the tablet together with the iron(II) chloride. The presence of amino acids alters the surface during the formation of iron silicate chemical gardens. The amino acids do not precipitate as crystalline solids but as an amorphous phase or isolated molecules.

In addition to physical aspects of our experiments related with the micromorphology, we have studied amino acid adsorption. Amino acid molecules can be adsorbed physically with weak interactions of electrostatic nature. But we have observed that the amino acid molecules are also interacting chemically, as shown in our spectroscopic results. Both amino acid molecules, arginine and tryptophan, are chemisorbed in all cases with the different procedures explored, with the amino acids adsorbed chemically to the iron cations of the chemical garden surfaces by hydrogen bonds and coordination complexes with the carboxylate groups. However, these chemical interactions are different in each case. In all cases, the carboxylic group of the amino acid is involved in its carboxylate form. The oxygen atoms of this group can form coordination complexes with the cations of the mineral surface in different forms, monodentate (only one oxygen), bidentate (both oxygen atoms) and combining with one or two cations simultaneously. At the same time, the carboxylate groups can form hydrogen bonds with the H atoms of the hydroxylated cations of the solid surface. All of these coordination possibilities can yield different profiles in the fine-structure of the IR spectra.

This research was undertaken having in mind the hypothesis that mineral surfaces were critical in the origin of prebiotic chemistry. These mineral surfaces provide confined spaces and absorption properties, where simple organic molecules can be adsorbed for later organic reactions to form more complex molecules. Our experiments show that the amino acids are adsorbed on the surface of these chemical gardens and alter the micromorphology of these surfaces. In contrast to our laboratory experiment time scales of minutes, on the early Earth these adsorptions could be produced during geological periods of time, probably combining adsorption–desorption processes offering opportunities for chemical reactions toward more complex molecules.

This work is one step toward showing how, if life first incubated within mineral membranes, for example within hydrothermal vents on the ocean floor of the early Earth, the hypothesis of a biological takeover of the self-assembled mineral structures with the incorporation of molecules of prebiotic chemistry such as amino acids is feasible. The first proto-cells could have manipulated the mineral membranes that formed their compartments in order to control their metabolism. This biomineralization process would have been the beginning of evolution, and with it, of biology.
